# CLIMATE RESILIENCE FOR AN AGING NATION

**DOI:** 10.1093/geroni/igae098.1006

**Published:** 2024-12-31

**Authors:** Nichole Kain, Danielle Arigoni

**Affiliations:** Antioch University New England, Saint Paul, Minnesota, United States; National Housing Trust, Washington, District of Columbia, United States

## Abstract

Our population is aging—by 2034, the US will have more people over 65 than under 18, and older residents make up a disproportionate number of casualties from natural disasters. In Climate Resilience for an Aging Nation, community resilience and housing expert Danielle Arigoni argues that we cannot achieve true resilience until communities adopt interventions that work to meet the needs of their oldest residents. Older adults are over-represented among fatalities in climate-fueled disasters, ranging from Hurricane Katrina in 2005 to the Maui wildfires of 2023 – and countless events in between -- people over 65 are significantly more likely to die as their younger counterparts. These rates of death have not improved in nearly 20 years, making it imperative that a renewed focus on climate resilience must be centered in the needs of older adults. This talk will provide an overview of how and why climate change differently impacts older people, and the range of solutions and strategies that can be deployed to reduce risk. Strategies include interventions in housing, transportation, emergency management, health care, utilities, and social infrastructure which can deliver a more climate-resilient community when designed with the needs of older adults in mind. With a role for people in all sectors and at all levels, achieving climate resilience for an aging nation truly is a job for all of us.

